# When “Metastatic Disease” Is Not Metastatic: Diffuse Large B-cell Lymphoma Presenting With a Perisplenic Mass, Pulmonary Masses, Pleural Effusion, and Retroperitoneal Lymphadenopathy

**DOI:** 10.7759/cureus.111802

**Published:** 2026-06-30

**Authors:** Rodrigo Furlan Silva Fabri, Liliana Penoth Gomez, Srija Sirineni, Abirami Balasubramanian, Vijay Chennareddy, Maha Soliman, Nawar Hakim, Barney Soskin

**Affiliations:** 1 Internal Medicine, Texas Tech University Health Sciences Center El Paso Paul L. Foster School of Medicine, El Paso, USA; 2 Pathology, Texas Tech University Health Sciences Center El Paso Paul L. Foster School of Medicine, El Paso, USA

**Keywords:** diffuse large b-cell lymphoma, extranodal lymphoma, hydronephrosis, mesenteric mass, metastatic mimic, pleural effusion, pulmonary masses, splenic lesion

## Abstract

Diffuse large B-cell lymphoma (DLBCL) is the most common aggressive non-Hodgkin lymphoma and may present with nodal or extranodal disease. Thoracic and abdominal extranodal involvement can produce a radiographic appearance that closely mimics disseminated metastatic solid-organ malignancy, particularly when pulmonary masses, pleural abnormalities, splenic lesions, and bulky abdominal disease coexist.

We report the case of an 81-year-old woman who initially presented with wheezing, productive cough, hypoxia, and dysuria and was referred for presumed pneumonia and urinary tract infection. Imaging subsequently revealed multiple bilateral pulmonary and pleural-based masses, bulky mediastinal and hilar lymphadenopathy, a large left upper quadrant/perisplenic mesenteric mass, splenomegaly with multiple splenic lesions, retroperitoneal and mesenteric lymphadenopathy, pleural effusion, and left hydroureteronephrosis due to malignant obstruction. Core needle biopsy of the left upper quadrant mesenteric mass demonstrated high-grade B-cell lymphoma (BCL) most compatible with diffuse large B-cell lymphoma. Immunohistochemistry showed cluster designation (CD)20, CD79a, CD10, and BCL6 positivity with negative BCL2, multiple myeloma oncogene1 (MUM1), and cyclin D1, consistent with a germinal center B-cell-like phenotype by the Hans algorithm, and Ki-67 was approximately 80%-90%. Flow cytometry identified a CD10-positive, kappa-restricted, abnormal mature B-cell population, and fluorescence in situ hybridization later demonstrated BCL2 rearrangement without MYC or BCL6 rearrangements, arguing against double-hit lymphoma.

This case highlights an important diagnostic pitfall: apparent widespread metastatic disease may in fact represent aggressive lymphoma, and early tissue diagnosis is essential when the radiographic pattern does not clearly fit a single solid-organ primary.

## Introduction

Diffuse large B-cell lymphoma (DLBCL) is a biologically heterogeneous lymphoma and remains the most common subtype of aggressive non-Hodgkin lymphoma in adults [1-3]. In routine practice, morphologic findings are integrated with immunohistochemistry and ancillary molecular studies, while cell-of-origin assignment is often approximated using immunohistochemical algorithms such as the Hans classifier when gene-expression methods are not available [1-8].

Extranodal lymphoma may involve the lung, pleura, spleen, mesentery, retroperitoneum, and urinary tract, and its imaging appearance may overlap substantially with infection, inflammatory disease, or metastatic carcinoma [3-8]. Pulmonary lymphoma can manifest as nodules, masses, mass-like consolidation, pleural disease, or mixed thoracic patterns, while mesenteric and retroperitoneal disease may appear as bulky soft-tissue masses and lymphadenopathy [4-8].

We describe an elderly woman whose initial evaluation suggested pneumonia and urinary tract infection, but whose imaging demonstrated diffuse thoracoabdominal disease interpreted as metastatic malignancy of unknown primary. Tissue diagnosis ultimately established high-grade B-cell lymphoma (BCL) most compatible with DLBCL, underscoring the importance of maintaining lymphoma in the differential diagnosis of apparently metastatic disease.

## Case presentation

An 81-year-old woman with a history of hypertension, anxiety, depression, remote pneumothorax in the 1970s, and urinary incontinence status post vaginal mesh and posterior repair presented after outside urgent care evaluation for presumed pneumonia and urinary tract infection. She reported approximately 10 days of wheezing followed by yellow sputum production and progressive cough with shortness of breath, in addition to dysuria. In the emergency department, she received ceftriaxone and azithromycin, but shortly after ceftriaxone administration, she developed hives and pruritus that improved with diphenhydramine. Initial chest radiography demonstrated right lower lobe mass-like consolidation, left lower lobe consolidation, and a left pleural effusion (Figure 1).

**Figure 1:**
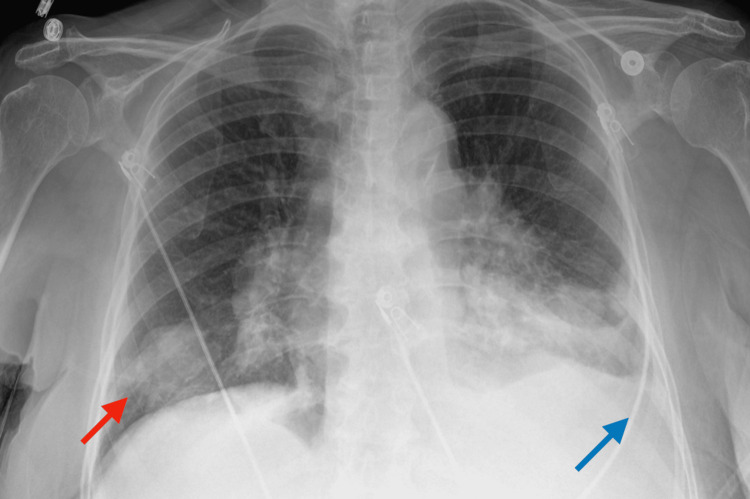
Initial chest radiograph showing right lower lobe mass-like opacity and left pleural effusion Anteroposterior chest radiograph obtained on presentation demonstrating a rounded mass-like opacity in the right lower lung base (red arrow), left basilar/retrocardiac airspace opacity, and a small left pleural effusion (blue arrow). These findings initially raised concern for pneumonia with an underlying malignant process.

Computed tomography (CT) of the chest with intravenous contrast showed multiple bilateral parenchymal and pleural-based pulmonary masses involving all lobes, the largest measuring 4.9 cm in the right lower lobe, together with bulky mediastinal and left hilar lymphadenopathy. Limited upper abdominal images revealed a 14.4 × 14.4 cm mass centered at the splenic hilum/perisplenic region, additional retroperitoneal nodal enlargement, and bilateral hydronephrosis. The overall radiographic impression strongly favored disseminated metastatic malignancy (Figures 2, 3).

**Figure 2:**
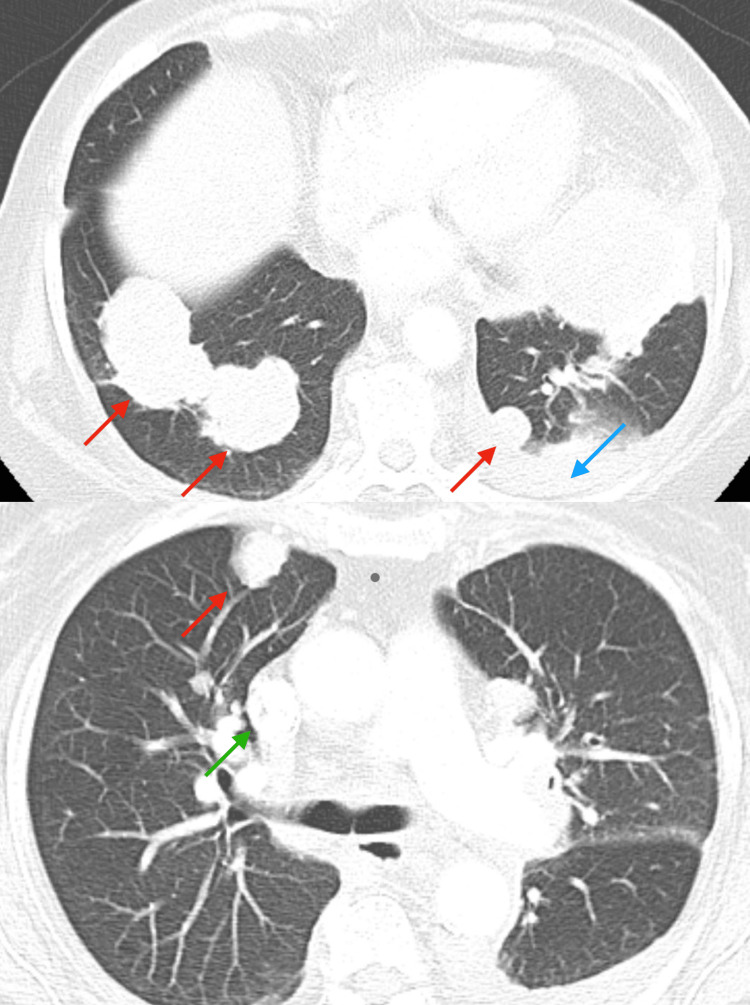
Axial CT chest images demonstrating multiple bilateral pulmonary masses, pleural-based disease, pleural effusion, and hilar lymphadenopathy Representative axial CT chest images demonstrating multiple bilateral pulmonary masses (red arrows), including pleural-based lesions, a small left pleural effusion (blue arrow), and right hilar lymphadenopathy (green arrow). The multiplicity and distribution of the thoracic lesions were initially highly suspicious for metastatic malignancy.

**Figure 3:**
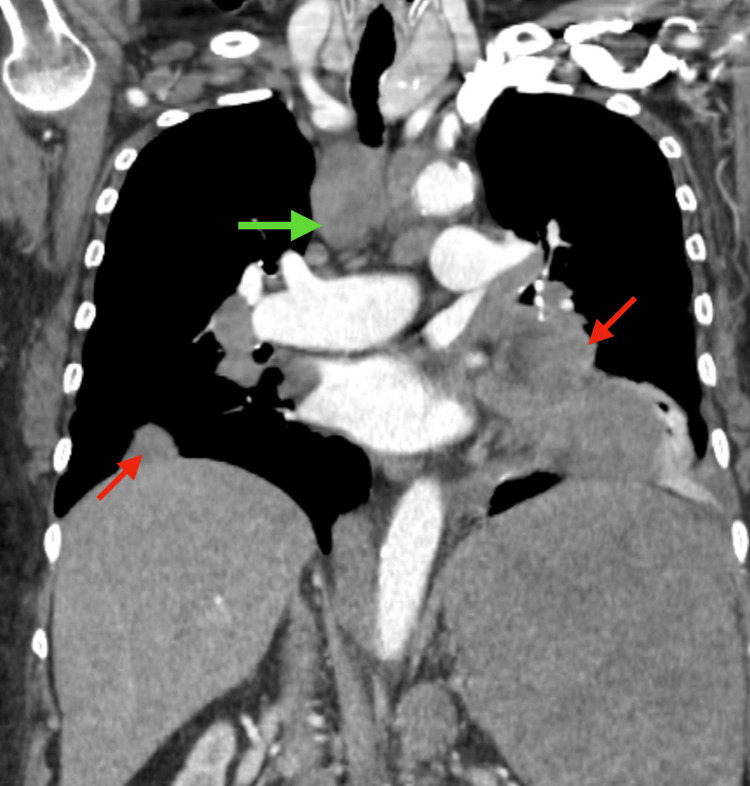
Coronal contrast-enhanced CT chest demonstrating bulky mediastinal and left hilar lymphadenopathy with bilateral thoracic masses Coronal contrast-enhanced CT image demonstrating bulky mediastinal lymphadenopathy (green arrow), a large left hilar/perihilar nodal mass (right red arrow), and an additional right basilar pleural-based pulmonary mass (left red arrow). The overall thoracic imaging pattern, including bulky mediastinal and hilar adenopathy with multiple masses, was initially considered highly suspicious for metastatic malignancy.

Further CT imaging of the abdomen and pelvis demonstrated splenomegaly measuring 18.5 cm with multiple splenic hypodense lesions, enlarged retroperitoneal and mesenteric lymph nodes, and moderate left hydroureteronephrosis attributed to mass effect from retroperitoneal lymphadenopathy. A partially visualized right pleural effusion and lower lung lesions were also noted. Tumor markers showed normal carbohydrate antigen 19-9 (CA 19-9) and carcinoembryonic antigen, with mildly elevated cancer antigen 125 (CA-125) at 44 U/mL. Additional history revealed approximately 30 pounds of unintentional weight loss over six months with decreased appetite (Figure 4).

**Figure 4:**
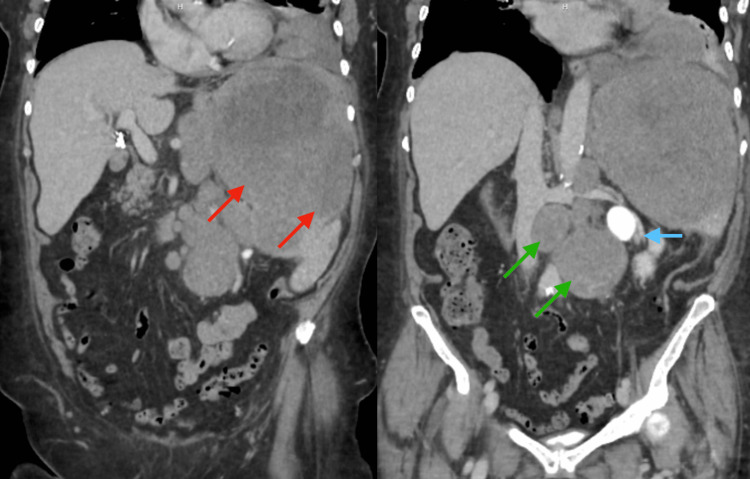
Coronal contrast-enhanced CT abdomen and pelvis demonstrating splenic involvement, bulky retroperitoneal/mesenteric lymphadenopathy, and left hydroureteronephrosis Representative coronal contrast-enhanced CT images of the abdomen and pelvis demonstrating marked splenomegaly with multiple splenic lesions (red arrows), bulky retroperitoneal and mesenteric lymphadenopathy including a dominant left para-aortic nodal mass (green arrows), and moderate left hydroureteronephrosis (blue arrow) secondary to mass effect from adjacent nodal disease. These abdominal findings further supported the initial radiographic impression of disseminated metastatic malignancy prior to tissue diagnosis.

Because of worsening obstructive uropathy and mild acute kidney injury, the patient underwent left nephrostomy tube placement, with subsequent decompression of the left collecting system on follow-up imaging. A moderate pleural effusion persisted, but bedside assessment later suggested insufficient fluid volume for thoracentesis. Brain magnetic resonance imaging showed only mild atrophy and chronic microvascular ischemic change without evidence of an intracranial mass lesion (Figure 5). Obstructive uropathy from malignant extrinsic compression is clinically important because prompt decompression may preserve renal function and improve symptoms.

**Figure 5:**
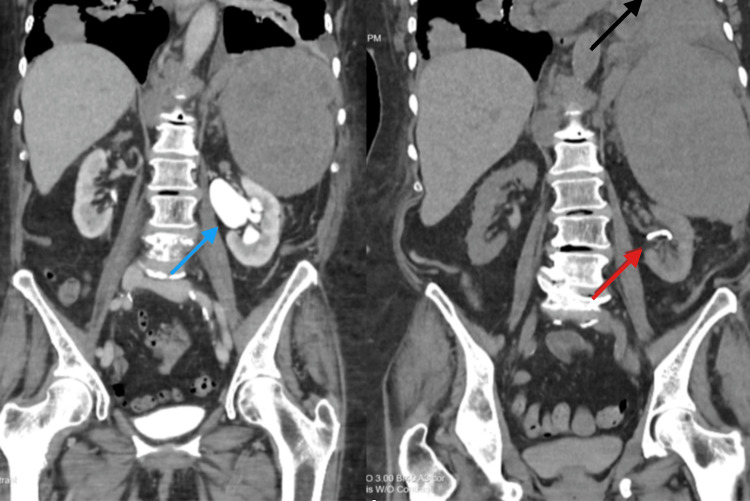
Coronal CT images demonstrating left hydroureteronephrosis before and decompression after percutaneous nephrostomy placement Paired coronal CT images of the abdomen and pelvis demonstrating moderate left hydroureteronephrosis before intervention (blue arrow, left panel) and decompression of the left renal collecting system after placement of a left percutaneous nephrostomy tube (red arrow, right panel). These findings illustrate relief of obstructive uropathy caused by mass effect from adjacent retroperitoneal nodal disease.

Ultrasound-guided needle core biopsy of the left upper quadrant mesenteric mass showed several cores containing a diffuse proliferation of medium- to large-sized atypical lymphoid cells with vesicular chromatin, small nucleoli, and numerous mitotic figures. Although morphologic preservation was described as suboptimal, the findings favored diffuse large B-cell lymphoma with high proliferative activity over another high-grade B-cell lymphoma (Figure 6).

**Figure 6:**
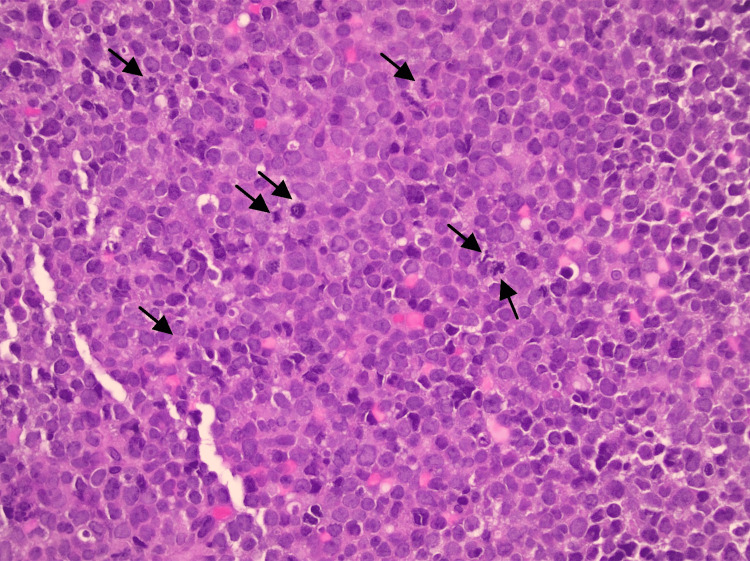
Mesenteric mass biopsy, H&E stain High-power hematoxylin and eosin section demonstrates a diffuse proliferation of medium- to large-sized atypical lymphoid cells with vesicular chromatin, small nucleoli, and frequent mitotic figures, including atypical mitoses highlighted by black arrows. The morphologic findings support diffuse large B-cell lymphoma with high proliferative activity. Original magnification ×400.

Immunohistochemistry showed the atypical lymphoid cells were positive for cluster designation (CD)20, CD79a, CD10, and BCL6 and negative for BCL2, multiple myeloma oncogene1 (MUM1), and cyclin D1. Ki-67 proliferative activity was approximately 80%-90%, and the pathology report interpreted this profile as fully consistent with a germinal center B-cell-like lymphoma by the Hans algorithm (Figures 7, 8). Immunohistochemical cell-of-origin assignment remains widely used in clinical practice, although gene-expression-based methods may provide more precise biologic subclassification.

**Figure 7:**
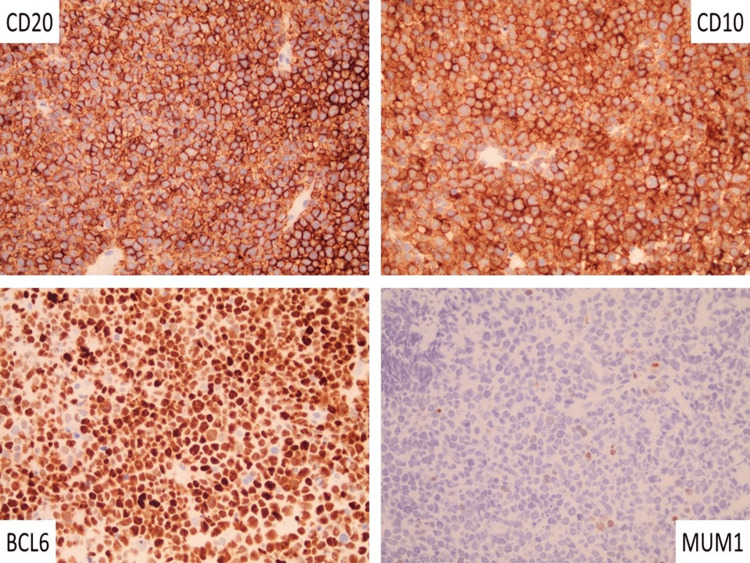
Immunohistochemistry supporting germinal center B-cell-like phenotype Representative immunohistochemical stains demonstrate strong tumor-cell positivity for CD20, CD10, and BCL6, with absence of staining for MUM1 (and cyclin D1, not shown). This immunophenotypic profile supports a germinal center B-cell–like (GCB) subtype of diffuse large B-cell lymphoma according to the Hans algorithm. Original magnification ×400.

**Figure 8:**
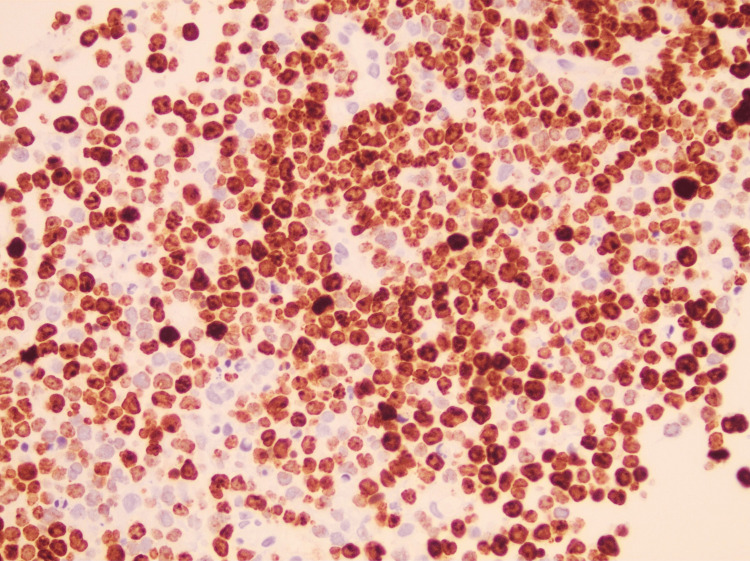
Ki-67 immunostain demonstrating high proliferative index Ki-67 immunohistochemical stain shows a markedly elevated proliferative index, with approximately 80%–90% of neoplastic lymphoid cells demonstrating strong nuclear positivity. This high proliferation fraction supports an aggressive large B-cell lymphoma. Original magnification ×400.

Flow cytometry identified an abnormal mature B-cell population comprising 49.9% of analyzed cells, with an aberrant immunophenotype including dim CD45 expression, CD19 positivity, CD5 negativity, CD10 positivity, Human Leukocyte Antigen-DR isotype (HLA-DR) positivity, CD38 positivity, CD20 positivity, partial CD79a, partial CD23, partial FMC7 expression, and kappa light-chain restriction. Surgical pathology confirmed a high-grade B-cell lymphoma, most compatible with diffuse large B-cell lymphoma. Immunohistochemistry demonstrated CD20, CD10, and BCL6 positivity, MUM1 negativity, and a high Ki-67 proliferative index, supporting a germinal center B-cell-like phenotype. Fluorescence in situ hybridization, performed externally, demonstrated a BCL2 rearrangement without MYC, BCL6, or IGH rearrangements. Therefore, the findings supported DLBCL and argued against MYC/BCL2 or MYC/BCL6 double-hit lymphoma. This distinction is clinically important because MYC-rearranged high-grade B-cell lymphomas are associated with different prognostic and therapeutic implications compared with conventional DLBCL.

## Discussion

This case is educational because the radiographic pattern initially suggested disseminated metastatic solid-organ malignancy rather than lymphoma. The combination of multifocal pulmonary and pleural-based masses, mediastinal adenopathy, pleural effusion, splenic lesions, a dominant perisplenic/mesenteric mass, and bulky retroperitoneal lymphadenopathy strongly resembled metastatic carcinoma of unknown primary. However, extranodal lymphoma can produce exactly this kind of misleading thoracoabdominal picture [3-8]. Pulmonary lymphoma has been described as presenting with nodules, masses, mass-like consolidation, pleural disease, and mixed radiographic patterns that overlap with infection or metastases [4-6].

The pathology in this case adds depth beyond a simple radiology-pathology correlation. Morphology favored DLBCL with high proliferative activity, while immunohistochemistry supported a germinal center B-cell-like phenotype by the Hans algorithm. The combination of CD10 and BCL6 positivity with MUM1 negativity fits a germinal center profile, but the later identification of BCL2 rearrangement raises the biologic possibility of transformed follicular lymphoma in the differential, even though the available core biopsy and final sign-out favored DLBCL rather than definitive transformed follicular lymphoma. That interpretation should be presented cautiously as a biologic possibility rather than a proven conclusion [1,2,9-17].

Another important teaching point is malignant obstructive uropathy. In this patient, bulky retroperitoneal disease caused left hydroureteronephrosis and mild acute kidney injury, requiring nephrostomy tube placement. Although obstructive uropathy is more often discussed in the setting of pelvic or retroperitoneal solid tumors, lymphoma-related ureteral obstruction is well described and should be recognized early because decompression can preserve renal function and support subsequent oncologic management [17-19].

Overall, this case supports a practical message: when imaging suggests “widespread metastatic disease,” but the pattern includes bulky lymphadenopathy, splenic lesions, and a dominant mesenteric or perisplenic soft-tissue mass, lymphoma should remain high in the differential diagnosis, and tissue confirmation should be obtained promptly [2-8]. Accurate pathologic classification is essential because staging, prognosis, and treatment differ substantially between DLBCL and other aggressive B-cell lymphomas [2,15,16]. Once the diagnosis is established, staging and response assessment should follow standard lymphoma frameworks such as the Lugano classification [19].

## Conclusions

Diffuse large B-cell lymphoma can closely mimic widespread metastatic solid-organ malignancy when it presents with multifocal pulmonary masses, pleural effusion, bulky perisplenic or mesenteric disease, splenic lesions, and retroperitoneal lymphadenopathy. This case emphasizes the importance of maintaining lymphoma in the differential diagnosis of apparent metastatic disease of unknown primary, particularly when bulky lymphadenopathy and splenic involvement are present. Prompt tissue diagnosis is critical because correct classification directly affects staging, prognosis, and management.
